# Headaches, Their Causes and Their Cure

**Published:** 1872-03

**Authors:** 


*Headaches, their Causes and their Cure.* By Henry G. Wright, M.D., M.R.C.S.L., L.S.A. From the Fourth London Edition. Philadelphia: Lindsay & Blakiston, 1871.

This work is in many respects of much value, and will not fail of being useful in the profession. It is divided into two parts. Part 1st being on the varieties and Symptoms of Headache. Part 2d on their Causes and Treatment. In treating of the Varieties of headache, the author speaks of the late suppers, late hours, and poorly ventilated rooms, of our fashionable balls and parties, as a prolific source of much suffering. Excessive study, or severe mental strain are also spoken of as well known causes. In the part on treatment, the removal of these causes are, in many instances, sufficient remedy, in others, some simple medicine is needed. The treatment which is laid down by the author in some cases is, to our mind, somewhat complex, and rather out of date. In many instances we believe that by Hygienic means alone, or with the aid of simple remedies, he might accomplish as much as he does with medicine..

